# Spatial Recognition Memory: Differential Brain Strategic Activation According to Sex

**DOI:** 10.3389/fnbeh.2021.736778

**Published:** 2021-09-01

**Authors:** Joaquín Castillo, Isabel Carmona, Sean Commins, Sergio Fernández, Juan José Ortells, José Manuel Cimadevilla

**Affiliations:** ^1^Department of Psychology, University of Almería, Almeria, Spain; ^2^Health Research Center, University of Almería, Almeria, Spain; ^3^Department of Psychology, Maynooth University, Kildare, Ireland

**Keywords:** spatial orientation, gender, spatial memory, dimorphism, evoked potential

## Abstract

Human spatial memory research has significantly progressed since the development of computerized tasks, with many studies examining sex-related performances. However, few studies explore the underlying electrophysiological correlates according to sex. In this study event-related potentials were compared between male and female participants during the performance of an allocentric spatial recognition task. Twenty-nine university students took part in the research. Results showed that while general performance was similar in both sexes, the brain of males and females displayed a differential activation. Males showed increased N200 modulation than females in the three phases of memory process (encoding, maintenance, and retrieval). Meanwhile females showed increased activation of P300 in the three phases of memory process compared to males. In addition, females exhibited more negative slow wave (NSW) activity during the encoding phase. These differences are discussed in terms of attentional control and the allocation of attentional resources during spatial processing. Our findings demonstrate that sex modulates the resources recruited to performed this spatial task.

## Introduction

Knowledge about the external world and how our brain uses this information during spatial orientation tasks have been the subject of many studies over the last decades. The development of computerized virtual reality (VR) tasks allows spatial abilities to be assessed in controlled environments while maintaining high levels of ecological validity (Matheis et al., 2007). A further advantage of VR tasks is the ability to combine them with different neuroimaging techniques, which are responsible for the identification of many neural structures underlying spatial behavior, such as the hippocampal area (Burgess et al., 2002), parietal (Husain and Nachev, 2007), and retrosplenial cortices (Mitchell et al., 2018), among others. Furthermore, the hippocampus and medial temporal lobe structures have been specifically implicated in allocentric representation (Iaria et al., 2009), the ability to form spatial associations between objects and locations that are independent of the viewer.

Because allocentric spatial orientation involves the medial temporal lobe, a brain region that also contributes to episodic memory (Burgess et al., 2002), these VR spatial allocentric tasks are especially interesting in the learning and memory research field. However, the combination of VR allocentric-based tasks and electrophysiological studies was not an easy job. Navigation in a virtual scenery demands different movements interfering with the collection of cortical activity. In addition, evoked brain activity requires that events be controlled in time, thus making possible to match behavioral and electrophysiological processes.

On this note, spatial recognition-based tasks are an attractive alternative method to assess allocentric processes, often requiring participants to retrieve memories from shifted viewpoints. Given that viewpoint manipulation has been reported to depend on the integrity of the medial temporal lobe (King et al., 2002; Lambrey et al., 2008), spatial recognition tasks could be appropriate paradigms to study the electrophysiological and neural features of allocentric spatial memory performance. Note that some previous studies used behavioral tasks demanding participants to decide about the position of an object or tray of objects on a blank background (Lithfous et al., 2014; Amico et al., 2015; Chueh et al., 2017), which could not be considered properly allocentric.

Moreover, spatial skills involve several competences some of them clearly sexually dimorphic (for a review see [Bibr B60][Bibr B15]). Regarding spatial recognition, previous studies reported that males outperformed females with better recognition rates (Ardila et al., 2011; Lojko et al., 2015; Tascón et al., 2016, 2017; Fernández-Baizán et al., 2018) or in certain spatial components like forming and using cognitive maps (Liu et al., 2011). However, females outperformed males in object location memory (Voyer et al., 2007; Bocchi et al., 2018). This sex-related performance could be mediated by differences in brain activity in regions like the hippocampus, where males are considered to have a more right lateralized activation than females (Frings et al., 2006; Persson et al., 2013), a fact that is overly related to better spatial performance. For an in-depth review on these hippocampal differences, see Yagi and Galea (2018).

Spatial recognition using EEG has been previously explored by Murphy et al. (2009), who found sex differences on parietal components (P300 at the CPz electrode site) for object recognition, with larger amplitudes for females. Their task design consisted in recalling the correct position of one stimulus when given various novel and studied viewpoints context free. The present study (adapted from Tascón et al., 2017), also examines EEG signals while participants performed a spatial recognition task with viewpoint manipulation. However, it examined the encoding of three stimuli in a complex environment, combining distal landmarks and perspective rotation, as well as the maintenance and the retrieval of this spatial information. As found in previous studies, higher difficulty levels are more prone to find sexual dimorphism in performance in favor of males, and, specially, viewpoint shifts favor the allocentric strategy and hippocampal involvement, thus preventing the use of egocentric solutions (Tascón et al., 2017). This also implies higher working memory demands, where male superiority tends to occur, as shown in a review by Coluccia and Louse (2004).

At the electrophysiological level, the role of the dorsal pathway in the processing of visuospatial information has been extensively studied, suggesting the involvement of attentional and memory processes (Ungerleider et al., 1998; Podzebenko et al., 2002). Ruchkin et al. (1996) found evidence that cortical streams recruited in visuospatial processing also contributed to storage and retention of such information. These streams were observed in parietal and occipital regions, remaining active throughout the entire encoding and maintenance intervals.

Previous research using visuospatial recognition paradigms found negative slow waves (NSW) in parietal and occipital areas related to retention of visuospatial information (Ruchkin et al., 1992, 1996; Mecklinger and Pfeifer, 1996; Carmona et al., 2020a) and also visuospatial manipulation (Liu et al., 2010; Riečanský et al., 2013). This NSW is usually preceded by the P300 component, a positive-going wave also detected in parietal and occipital regions peaking about 350–550 ms after the stimuli onset (Ruchkin et al., 1996; Roalf et al., 2006; Carmona et al., 2020a) and related to the detection and discrimination of relevant stimuli (Polich, 2012; Huang et al., 2015). There was also described a N200 component, a negative-going wave in central and parietal sites peaking about 250–350 ms after the stimuli onset and related to cognitive control processes (Potts, 2011; Koivisto et al., 2018; Carmona et al., 2020a).

As there has been little work examining sex differences using EEG and spatial recognition (see Murphy et al., 2009), in this experiment we explored the electrophysiological correlates underlying the performance of this allocentric spatial recognition task, using the event related potentials (ERP) technique. We explored sex-dimorphic patterns since males and females could implement various strategies (Roalf et al., 2006; Hirnstein et al., 2019) or show some visuospatial processing differences (for a review, see Vanston and Strother, 2017).

According to the aforementioned researches, we expected to find different behavioral and electrophysiological profiles in males and females. Regarding to the behavioral data, it is hypothesized that males could outperform females in correct responses, as demonstrated by Tascón et al. (2017). Moreover, at electrophysiological level, it is predicted that sex differences could be observed in the NSW, since this wave is related to maintenance and manipulation of visuospatial information. P300 component could also reflect sex differences as described in previous research on spatial memory (Murphy et al., 2009).

## Materials and Methods

### Participants

Twenty-nine participants, sixteen females (*X* = 23.5; SD = 2.30) and thirteen males (*X* = 24.35; SD = 2.20), all of them students from the University of Almería, voluntarily took part in the study. They had normal or corrected vision in the moment of the assessment. Exclusion criteria were addressed in a brief initial interview, which screened for any psychological or psychiatric disorder, drug, tobacco and alcohol abuse, head traumatisms, or similar issues that could influence cognitive performance. The study was approved by the University of Almería Ethical Committee and fulfills the requirements of the European Communities Council Directive 2001/20/EC. *Post hoc* power calculations were conducted with the G^∗^Power software, version 3.1.9.2 (Faul et al., 2007) in order to determine the minimum statistical power of both main and interaction effects (within-between-subject factors) showed in our study. With an alpha = 0.05, a medium effect size (*d* = 0.42) and total sample size = 29, the analysis revealed statistical power greater than 0.99.

### Procedure

Evaluations were run individually for each participant in a quiet laboratory setting free from noise and distractions. All signed an informed consent document and underwent an initial interview to pinpoint the exclusion criteria before the commencement of the experiment.

**FIGURE 1:**
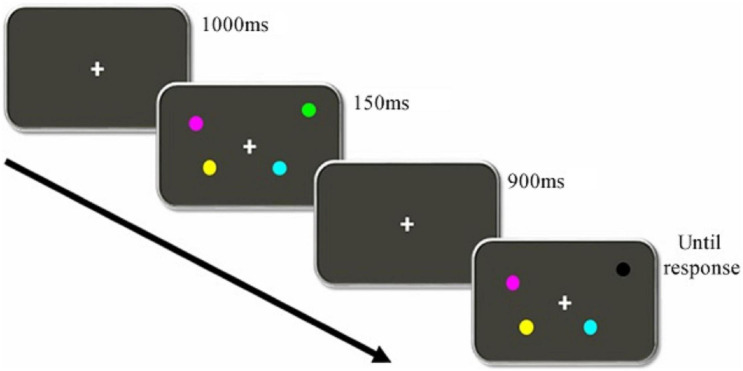
Sequence of events for a Change Location Task trial. Participants needed to identify the circle that changed colors between presentations.

In order to assess the working memory capacity (WMC) at a behavioral level (no EEG registry), the Change Localization Task (Johnson et al., 2013; Noguera et al., 2019; Castillo et al., 2020; Fernández et al., 2021) was used. The task was designed using the e-Prime 2.0 software (Psychology Software Tools). Figure 1 represents the sequence of events presented in each trial.

At first, a fixation point was shown for 1,000 ms. Directly afterward, four colored circles were displayed for 150 ms. Minimal radius from the fixation point to the closest stimulus was 3.36° and 6.24° for the farthest. Possible colors for the circles (with the RGB values) were orange (255, 113, and 0), green (0, 255, and 0), yellow (255, 255, and 0), cyan (0, 255, and 255), magenta (255, 0, and 255), blue (0, 0, and 255), red (255, 0, and 0), white (255, 255, and 255) and black (0, 0, and 0), without repeating colors for a single trial. Then, after a 900 ms delay, another set of four circles was presented, whose colors and positions were the same as those in the previous set except for one that was colored differently. Participants had to click on the left click button of a laptop mouse to choose the circle which had changed its color between presentations.

An initial practice block, consisting of eight trials, was provided to each participant, along with visual feedback following each trial. Participants were then required to perform two consecutive experimental blocks of 32 trials each, with a short break between each block to avoid fatigue. Full task duration was around 8–10 min. Mean correct response scores for both blocks were combined and transformed to a *k*-index, based on the Pashler-Cowan equation (see Cowan et al., 2005). This index allows to identify the number of items present in WM using false alarms and hit rates. The proportion of correct responses for the 64 valid trials was multiplied by four (equaling the number of circles per trial) to obtain the *K*-Index as the WMC reference. This equals the mean number of colored circles a participant can memorize in the task, with *k* = 1 or 25% being the chance level.

**FIGURE 2:**
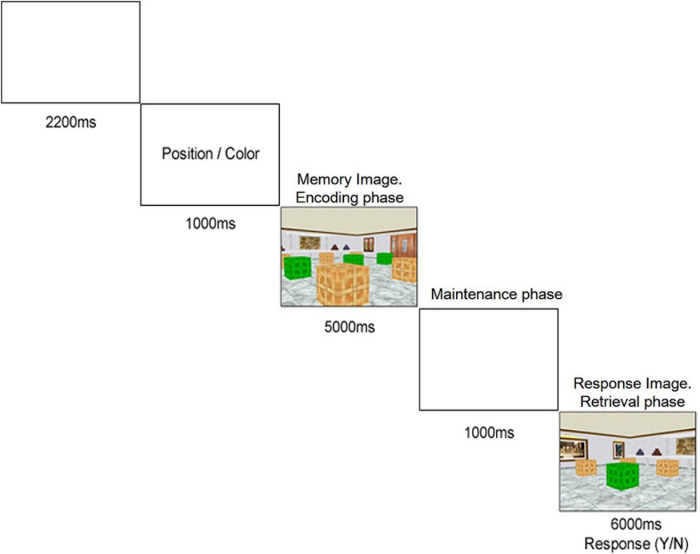
Example of a trial sequence for the spatial recognition task. Depending on the Criteria, participants need to memorize different information of a Memory Image and subsequently answer about it in a Recognition Image.

To examine spatial memory performance concurrently with ERP technique, a virtual 2D recognition test was used, based on the 3D environment of The Boxes Room and the procedure (Cánovas et al., 2008) and 2D stimuli of the Almeria Spatial Memory Recognition Test (ASMRT, Tascón et al., 2017). ASMRT design was adapted based on the recommendations of Woodman (2010) regarding electrophysiological studies with evoked potentials (ERPs). The sequence of events per trial is represented in Figure 2.

At the beginning of each trial, after a 2,200 ms delay, a word (in black font, 18 size and Times New Roman style) was presented for 1,000 ms at the center of the screen, considered the “Criteria.” This word could be one of two possible alternatives: Position or Color. Directly afterward, an image of a room including a series of nine boxes placed on a 3 × 3 disposition grid (the “Memory Image”) was presented to the participants for 5,000 ms. Three of those boxes were colored green or red instead of the regular brown, and depending on the Criteria, the information demanded to memorize from them was different:

• If the Criteria were “Position,” participants had to memorize the places occupied by the three outlined boxes in the room, regardless if they are red or green.
• If the Criteria were “Color,” participants had to retain the color (green or red, all of them identically colored) of those three outlined boxes in the room, regardless of their position in the museum room.

After a brief delay of 1,000 ms, another image of the same room (the “Recognition Image”) was presented until participant response, for a maximum of 6,000 ms. This time, only one of the boxes was colored green or red, and participants were demanded to answer if it matched the criteria from the previously memorized ones. Using an USB game controller, they should press the left trigger if their answer was positive (“yes”) and the right trigger if it was negative (“no”). Responses were based on the previous Criteria:

• For the “Position” criteria, participants needed to answer if the outlined box of the Recognition Image was in the same position that one of the three from the Memory Image, regardless if they matched colors.
• For the “Color” criteria, participants needed to answer if the outlined box of the Recognition Image matched the color of the three boxes from the Memory Image, regardless if position was the same or not.

The task consisted of an initial practice block of eight trials supervised by the experimenter, followed by a total of 128 experimental trials. Trials were separated in two blocks (64 trials each), with a brief pause in the middle. Each block consisted of 32 unique pairs of memory and recognition images, which were presented twice (one for each criteria), and randomized. The viewpoint was different in memory image and in recognition image. The angle of rotation between each pair of images was always the same, for color and position trials. Percentage of correct responses (correct acceptances and correct rejections) and reaction times were registered.

Finally, electroencephalographic (EEG) data for each participant were recorded for the full duration of the experimental blocks of the spatial task using a Brain Products actiCAP helmet, with 30 scalp channels following the international 10–10 system. This was coupled with a compatible Brain Products AC-amplifier in order to digitize the signals, with a sampling frequency of 250 Hz (0.1–70 Hz band-pass, 50 Hz notch filter), digitally band-pass filtered (high cutoff: 25 Hz, 24 dB/octave attenuation; low cutoff: 0.1 Hz, 12 dB/octave attenuation). A midfrontal electrode (FCz) was used as the reference channel, with the ground electrode placed between Fpz and Fz. Two additional electrodes were placed to record vertical (VEOG) and horizontal (HEOG) electrooculograms. Lastly, another set of two electrodes were situated on left and right mastoid locations in order to a posterior re-reference of EEG data. Impedance at all electrodes remained below 5 kΩ. Assessment was performed in an isolated room that mitigated electrical noise which could alter the registry.

Electroencephalographic markers were placed in the spatial task design to facilitate differentiation between codification and recovery memory processes, and differentiated due to different components of task methodology. This allowed to classify trials for subsequent analyses due to different variables such as Criteria, Decision Type or Response Type.

Independent component analyses (ICA; Makeig et al., 1997) were used to correct EEG data for ocular/blink artifacts. Then, the corrected data of the spatial task were segmented from: (i) encoding phase: 200 ms pre Memory Image onset to 5,000 ms post Memory Image onset; (ii) maintenance phase: 200 ms pre Memory Image offset to 1,000 ms post Memory Image offset; and (iii) retrieval phase: 200 ms pre Response Image onset to 1,500 ms post Response Image onset. Only trials with correct responses were included in the segmentations. Later on, EEG were corrected to a 200 ms baseline before the start of each segment (i.e., the last 200 ms of the preceding screen). Artifacts in each EEG segment and channel were rejected automatically (maximal allowed amplitude ±100 μV; maximal allowed voltage step 50 μV; maximal allowed difference of values in intervals 200 μV; lowest allowed activity 0.5 μV, interval length 100 ms). EEG data were re-referenced to averaged mastoids before the segments were averaged. The number of averaged segments was greater than 40 (>50% of valid trials) in all conditions, there were no significant differences in valid trials between conditions.

Three regions of interest (ROI), central (C), parietal (P), and occipital (O), were examined in order to explore N200 (Koivisto et al., 2018), P300 (Roalf et al., 2006; Steffensen et al., 2008), and NSW (Liu et al., 2010; Riečanský et al., 2013) components. A bilateral electrode pairs and a middle electrode were selected in each ROI (C: C3, Cz, and C4; P: P3, Pz, and P4; and O: O1, Oz, and O2). Additionally, *t*-tests were conducted between the male and female group at each electrode (30) for criteria, in time windows of 100 ms, with the aim of establishing the most appropriate time intervals and cluster of electrodes for component analysis.

### Data Analysis

For EEG data processing, the Brain Vision Analyzer 2.0 software was used. Processing of behavioral task performance alongside EEG data was analyzed using IBM SPSS Statistics 25, with a confidence level of *p* < 0.05.

#### Statistical Analyses

Behavioral data (accuracy and reaction times for correct choices), were analyzed with a mixed analysis of variance (ANOVA) with Sex (Male vs. Female) as the between-subject factor, Blocks (Block 1 and Block 2), Decision Type (Acceptance vs. Rejection) and Criteria (Color vs. Position) as the within subject factors (2 × 2 × 2 × 2).

Electrophysiological data were analyzed with mixed ANOVAs with Sex (Male vs. Female) as the between subject factor, Criteria (Color vs. Position), Decision Type (Acceptance vs. Rejection, only in the retrieval phase), Laterality (Left, Middle, and Right), and Caudality (C, P, and O) as the within subject factors (2 × 2 × 2 × 3 × 3), and ERP components in ROI (average voltage data in each time windows).

Kolmogorov-Smirnov tests were conducted to check normality of data, and Levene’s tests to verify homogeneity of variance. Bonferroni correction was applied to correct for type I error accumulation in multiple comparisons.

#### *T*-Tests Analyses

*T*-test were conducted in order to identify significant differences between ERPs signals in the NSW time window and the baseline (zero value) in ROI. Also, *T*-tests were conducted as complementary behavioral analyses.

#### Correlation Analyses

Correlation analyses were performed by using the Pearson’s correlation coefficient, in order to evaluate the relationship between behavioral measures of performance (correct responses and reaction times) and *k*-scores. Only significant correlations (*p*s < 0.05) are reported in the “Results” section.

## Results

### Behavioral Results

#### Accuracy Data

**FIGURE 3:**
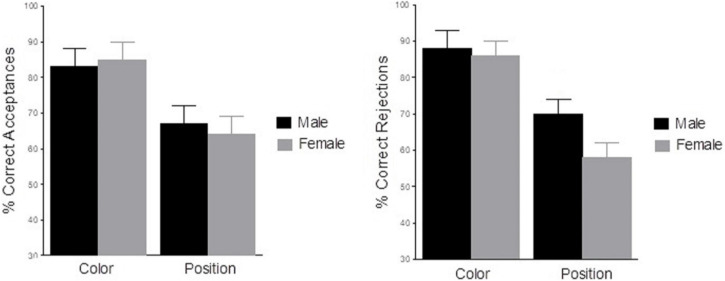
Mean percentage of correct responses (*left graph*, Hits; *right graph*, Correct Rejections) as a function of Criteria (Color vs. Position) and Sex (Men vs. Women). Error bars represent the standard deviations.

The analysis of correct responses (see Figure 3) revealed a significant main effect of Criteria [*F*(1,27) = 31.9, *p* < 0.001, ηp^2^ = 0.54], indicating that participants were less accurate in Position trials (*X* = 63%, SD = 0.03) than in Color trials (*X* = 86%, SD = 0.03). No other main effects or interactions were statistically significant (*p* > 0.05).

Complementary *T*-test analyses showed that there were no significant differences in correct rejections due to Sex [*t*(27) = 2, *p* = 0.055; mean 70 vs. 63%, male vs. female)] in Position trials as can be seen in Figure 3.

**FIGURE 4:**
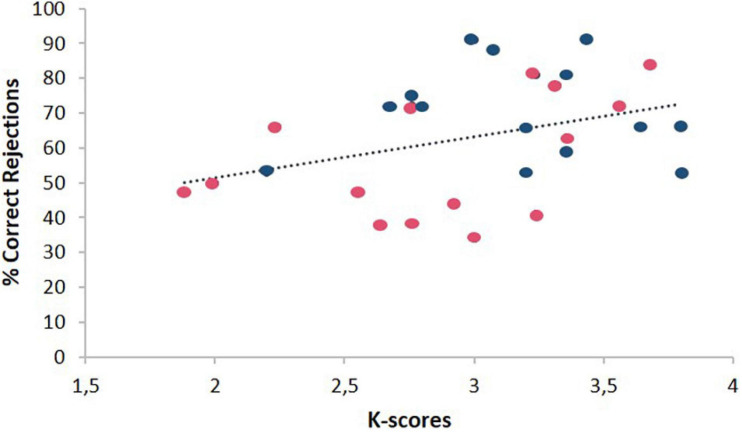
Dispersion of mean percentage of Correct Rejections in Position trials in the Spatial Memory Task due to the K-index. Blue point (male), red point (female).

Also, as can be seen in Figure 4, complementary analyses showed a positive significant correlation between mean percentage of correct rejections in Position trials and *k*-scores (*N* = 29; *r* = 0.37, *p* = 0.03). No other significant correlations nor difference were found (*p* > 0.05).

The analysis of reaction times (of correct choices, correct acceptances and correct rejections) showed a main effect of Block [*F*(1,27) = 10.7, *p* = 0.003, ηp^2^ = 0.28], Criteria [*F*(1,27) = 76.5, *p* < 0.001, ηp^2^ = 0.74], and Decision Type [*F*(1,27) = 7.9, *p* = 0.011, ηp^2^ = 0.21]. Participant’s response times were slower in the first block (*X* = 1,952 ms; SD = 51) than in the second block (*X* = 1,783 ms; SD = 53); their responses were faster in the Color criteria (*X* = 1,365 ms; SD = 115) than in the Position criteria (*X* = 2,374 ms; SD = 115); finally, they were slower to respond to rejection trials (*X* = 1,946 ms; SD = 83) than to acceptance trials (*X* = 1,789 ms; SD = 86).

**FIGURE 5:**
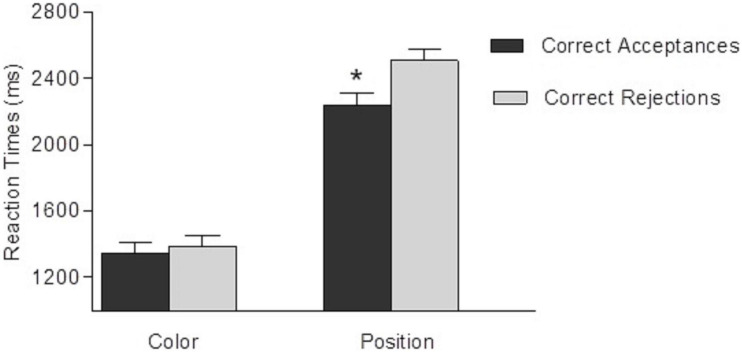
Mean of reaction times for correct choice, as a function of Criteria (Color vs. Position) and Decision Type (Hits vs. Correct Rejections). Error bars represent the standard deviations. Simple *p-value = 0.001.

Interaction Criteria *X* Decision Type was found statistically significant [*F*(1,27) = 7.6, *p* = 0.011, ηp^2^ = 0.08]. The analysis of the interaction showed a significant effect of Decision Type in Position trials [*F*(1,27) = 13, *p* = 0.001, ηp^2^ = 0.33], indicating that reaction times (Figure 5) were slower (*p* < 0.05) in correct rejection trials (*X* = 2,506 ms; SD = 51) than in correct acceptance trials (*X* = 2,235 ms; SD = 83). In contrast, Decision Type effect was not found in Color trials (*X* = 1,386 ms, SD = 74, and *X* = 1,342 ms, SD = 74, rejection and acceptance trials, respectively) [*F*(1,14) = 0.41, *p* = 0.511, ηp^2^ = 0.02]. No other effect nor interactions were statistically significant (*p* > 0.05).

### Electrophysiological Data

#### Encoding Phase

##### N200: time window from 250 to 350 ms after the memory image onset

The analysis revealed a significant main effect of Sex [*F*(1,27) = 7.6, *p* = 0.010, ηp^2^ = 0.22] and Caudality [*F*(2,26) = 17, *p* < 0.001, ηp^2^ = 0.57]. A higher wave deflection was registered in the male group (*X* = 0.93 mV, SD = ±0.63) than in the female group (*X* = 3.26 mV, SD = ±0.56); and wave deflection was greater in O region (*X* = 2 mV, SD = ±0.58) than in P region (*X* = 2.5 mV, SD = ±0.46) and in C region (*X* = −0.06 mV, SD = ±0.51). No other main effects nor interactions were found (see Table 1).

**TABLE 1:** Main significant results from analysis of variances (ANOVAs) on event related potentials (ERPs) amplitudes in the three phases (*f*: effect size; *p*, significance in brackets after Bonferroni correction, simple *p < 0.05; **p < 0.01; and ***p < 0.001).

**Phase**	**Time window *(ms)***	**ERP component**	***f* from ANOVAs**		
				
			**Sex**	**Criteria**	**Interaction effects**
Encoding	250–350	N200	0.53^(^**^)^	n.s.	n.s.
	350–550	P300	0.45^(^*^)^	n.s.	Sex × Caudality 0.55^(^*^)^
	1,000–5,000	NSW	n.s.	1.3^(^**^)^	Criteria × Caudality 0.75^(^*^)^
Maintenance	250–350	N200	0.96^(^***^)^	n.s.	Sex × Caudality 0.42^(^*^)^
	350–550	P300	0.44^(^*^)^	n.s.	Sex × Caudality 0.59^(^*^)^
Retrieval	250–350	N200	0.50^(^*^)^	0.44^(^*^)^	Sex × Caudality 0.42^(^*^)^
	350–550	P300	0.44^(^*^)^	0.61^(^*^)^	Sex × Caudality 0.59^(^*^)^ Decision Type × Laterality 0.36^(^*^)^

##### P300: time window from 350 to 550 ms after the memory image onset

**FIGURE 6:**
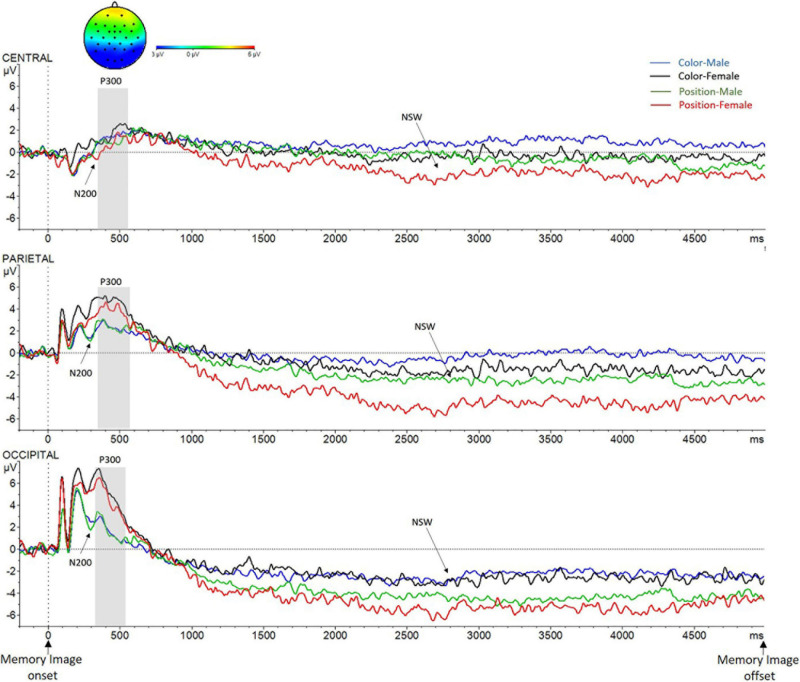
Grand-average voltage data (in mV) of ERP in the Encoding phase in ROI, as a function of Criteria and Sex (Color_Men, blue line; Color_Women, black line; Position_Men, green line; and Position_Women, red line). Gray shades represent the P300 time window. On the top, topographic map of the difference in ERP waves due to Sex (Men–Women) in the P300 time window. NSW, negative-slow wave. Time zero represents Memory Image onset.

The ANOVA showed a significant main effect of Sex [*F*(1,27) = 5.5, *p* = 0.027, ηp^2^ = 0.17], with an increased electrical activity being recorded in the female group (*X* = 3.4 mV, SD = ±0.95) compared to the male group (*X* = 1.2 mV, SD = ±0.93); and Caudality [*F*(2,26) = 24.4, *p* < 0.001, ηp^2^ = 0.65]. A greater activity in P region (*X* = 3.12 mV, SD = ±0.53) than in O region (*X* = 2.6 mV, SD = ±0.63) and in C region (*X* = 1.1 mV, SD = ±0.54) was registered (see Figure 6). Sex *X* Caudality interaction effect also reached significance [*F*(2,26) = 3.92, *p* = 0.032, ηp^2^ = 0.23]. The interaction analysis showed a main effect of Sex in P region [*F*(1.27) = 6.3, *p* = 0.021, ηp^2^ = 0.19; males (*X* = 1.8 mV, SD = ±0.79), females (*X* = 4.5 mV, SD = ±0.72)] and in O region [*F*(1,27) = 7.6, *p* = 0.01, ηp^2^ = 0.22; males (*X* = 0.91 mV, SD = ±0.93), females (*X* = 4.4 mV, SD = ±0.84)], but not in C region (*p* > 0.05). No other main effect nor interactions were found (*p* > 0.05).

##### NSW: time window from 1,000 to 5,000 ms after the memory image onset

The analysis revealed a significant main effect of Criteria [*F*(1,27) = 9, *p* = 0.006, ηp^2^ = 0.625] and Caudality [*F*(2,26) = 17, *p* < 0.001, ηp^2^ = 0.60]. Wave amplitude in Position trials (*X* = −2.8 mV, SD = ±0.59) was significantly more negative than in Color trials (*X* = −1.1 mV, SD = ±0.50). Activity in O region (*X* = −3.4, SD = ±0.66) was more negative than it was in both P region (*X* = −1.9, SD = ±0.49) and C region (*X* = −0.4 mV, SD = ±0.48). The Criteria *X* Caudality interaction effect also reached significance [*F*(2,26) = 7.2, *p* = 0.003, ηp^2^ = 0.36]. The interaction analysis revealed a significant main effect of Criteria in each of the three regions [C: *F*(1,27) = 5.1, *p* = 0.032, ηp^2^ = 0.16; P: *F*(1,27) = 12.4, *p* = 0.002, ηp^2^ = 0.32; and O: *F*(1,27) = 7.4, *p* = 0.011, ηp^2^ = 0.22], in all of them less amplitude was registered in color trials (C: *X* = −0.18 mV, SD = ±0.57; P: *X* = −0.86 mV, SD = ±0.54; and O: *X* = −2.5 mV, SD = ±0.60) than in position trials (C: *X* = −0.98 mV, SD = ±0.51; P: *X* = −3.0 mV, SD = ±0.62; and O: *X* = −4.4 mV, SD = ±0.87). No other significant effects nor interactions were found (*p* > 0.05).

Although the interaction Criteria *X* Caudality *X* Sex was not found [*F*(2,26) = 1.4, *p* = 0.26, ηp^2^ = 0.10], complementary *T*-test analyses (see Table 2) showed statistically significant differences in the female group due to criteria in the three ROI. [C: *t*(15) = 2.2, *p* = 0.049; P: *t*(15) = 3.2, *p* = 0.007; and O: *t*(15) = 2.4, *p* = 0.032]. In contrast, there were not differences in the male group in no region (ps > 0.05). In addition, differences between males and females were found in the NSW amplitude only for position trials in parietal region [*t*(27) = 2.1, *p* = 0.041], females exhibited a reliable higher wave amplitude than males (as can be seen in Figure 6). No other significant differences were found (*p* > 0.05).

**TABLE 2:** *p*-value obtained by comparing the electrical activity in the negative slow wave (NSW) time window: (a) with the baseline by region of interest (Central, Parietal, and Occipital), criteria (color, position) and sex (men, women); (b) between color and position trials by ROI and sex; (c) between sexes, by ROI and criteria.

**R.O.I.**	**(a) *T*-test (NSW vs. Baseline)**				**(b) *T*-test (Color vs. Position)**		**(c) *T*-test (Men vs. Women)**	
	**Men**		**Women**		**Men**	**Women**	**Color**	**Position**
	**Color**	**Position**	**Color**	**Position**				
**Central**	n.s.	n.s.	n.s.	*	n.s.	*	n.s.	n.s.
**Parietal**	n.s.	*	n.s.	***	n.s.	**	n.s.	*
**Occipital**	**	**	*	**	n.s.	*	n.s.	n.s.

Simple *p < 0.05; **p < 0.01; and ***p < 0.001.

Finally, we compared the sustained NSW with the baseline in each of the conditions per group (see Table 2). These *T*-tests analyses showed that in the female group, the NSW was significantly different from baseline (0 value) in position trials, in the three regions. In contrast, in the male group, the NSW was similar to baseline in position trials in C region.

#### Maintenance Phase

##### N200: time window from 250 to 350 ms after the memory image offset (delay period)

**FIGURE 7:**
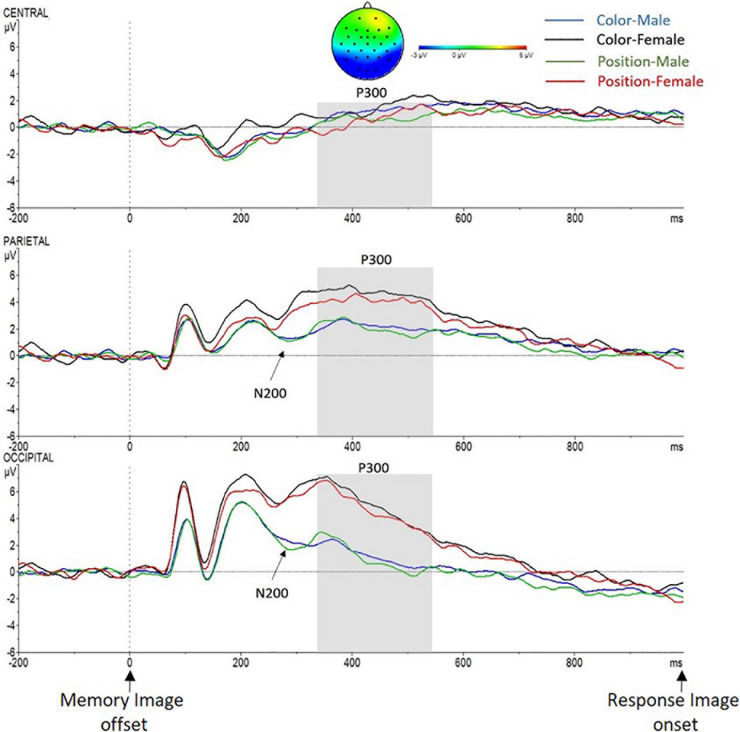
Grand-average voltage data (in mV) of ERP in the Maintenance phase (delay period) in ROI, as a function of Criteria and Sex (Color Men, blue line; Color Women, black line; Position Men, green line; and Position Women, red line). Gray shades represent the P300 time window. On the top, topographic map of the difference in ERP waves due to Sex (Men–Women) in the P300 time window. Time zero represents Memory Image offset.

Significant main effects of Sex [*F*(1,27) = 24.6, *p* < 0.001, ηp^2^ = 0.48] and Caudality [*F*(2,26) = 28.6, *p* < 0.001, ηp^2^ = 0.51] were found. Again, as in the N200 time window in the encoding phase, a higher wave deflection in the male group (*X* = 0.98 mV, SD = ±0.65) compared to the female group (*X* = 3.2 mV, SD = ±0.59) was found; also, wave deflection was greater in O region (*X* = 2.6 mV, SD = ±0.53) than in P region (*X* = 2.5 mV, SD = ±0.49) and in C region (*X* = −0.16 mV, SD = ±0.53). Similar to the encoding phase, Sex *X* Caudality interaction reached significance [*F*(2,26) = 4.8, *p* = 0.012, ηp^2^ = 0.15]. The interaction analyses showed a main effect of Sex in P region [*F*(1.27) = 6, *p* = 0.021, ηp^2^ = 0.18; male (*X* = 1.3 mV, SD = ±0.73), female (*X* = 3.7 mV, SD = ±0.66)] and in O region [*F*(1,27) = 10.8, *p* = 0.003, ηp^2^ = 0.29; male (*X* = 1.6 mV, SD = ±0.73), female (*X* = 4.1 V, SD = ±0.64)], but not in C region (*p* > 0.05). No other main effects nor interactions were found (*p* > 0.05; see Figure 7).

##### P300: time window from 350 to 550 ms after the memory image offset

As in the encoding phase, a significant main effect of Sex [*F*(1,27) = 5.2, *p* = 0.031, ηp^2^ = 0.16] was found in the P300 time window, and again, increased amplitude was recorded in the female group (*X* = 3.5 mV, SD = ±0.65) compared to the male group (*X* = 1.3 mV, SD = ±0.74); and Caudality [*F*(2,26) = 27.5, *p* < 0.001, ηp^2^ = 0.68], a greater activity in P region (*X* = 3.2 mV, SD = ±0.55) and in O region (*X* = 3.4 mV, SD = ±0.63) than in C region (*X* = 0.68 mV, SD = ±0.54) was registered (see Figure 7). A Sex *X* Caudality interaction effect was also found [*F*(2,26) = 4.7, *p* = 0.021, ηp^2^ = 0.26]. As in the previous phase, the interaction analysis showed a main effect of Sex in P region [*F*(1.27) = 5.3, *p* = 0.029, ηp^2^ = 0.16; males (*X* = 1.9 mV, SD = ±0.81), females (*X* = 4.5 mV, SD = ±0.73)] and in O region [*F*(1,27) = 9.9, *p* = 0.001, ηp^2^ = 0.27; males (*X* = 1.4 mV, SD = ±0.93), females (*X* = 5.3 mV, SD = ±0.84)], but not in C region (*p* > 0.5). No other main effects nor interactions were found.

#### Retrieval Phase

##### N200: time window from 250 to 350 ms after the response image onset

**FIGURE 8:**
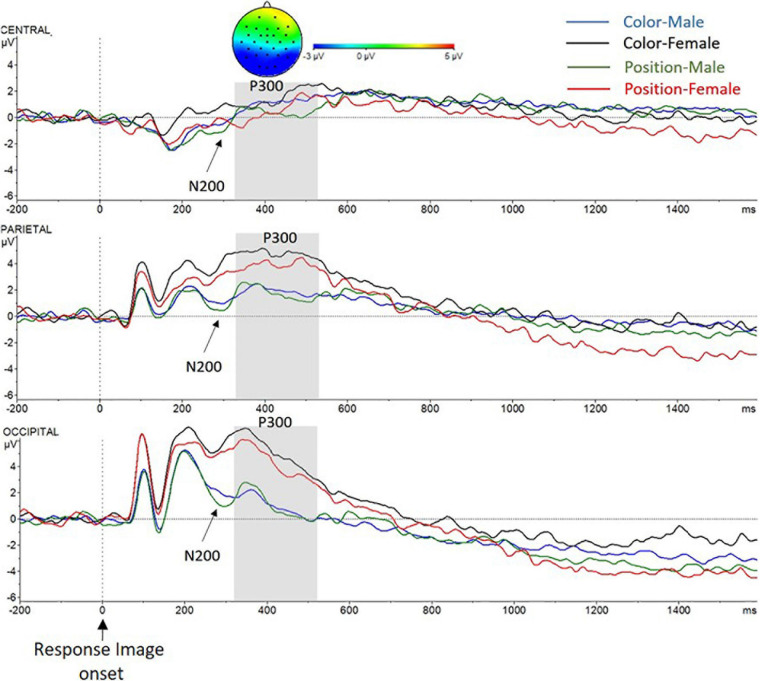
Grand-average voltage data (in mV) of ERP in the Retrieval phase in ROI, as a function of Criteria and Sex (Color Men, blue line; Color Women, black line; Position Men, green line; and Position Women, red line). Gray shades represent the P300 time window. On the top, topographic map of the difference in ERP waves due to Sex (Men–Women) in the P300 time window. Time zero represents Response Image onset. P300. Time window from 350 to 550 ms after the Response Image onset.

Examination of the retrieval phase also revealed a significant main effect of Sex [*F*(1,27) = 7.3, *p* = 0.012, ηp^2^ = 0.21] and Caudality [*F*(2,26) = 20.4, *p* < 0.001, ηp^2^ = 0.60]. Again, as in the N200 time window in the encoding and in the maintenance phases, higher wave deflection was found in the male group (*X* = 0.86 mV, SD = ±0.64) compared to the female group (*X* = 3.1 mV, SD = ±0.57); also, wave deflection was greater in O region (*X* = 2.5 mV, SD = ±0.59) than in P region (*X* = 2.4 mV, SD = ±0.47) and in C region (*X* = −0.16 mV, SD = ±0.52), as can be seen in Figure 8. A Sex *X* Caudality interaction was found [*F*(2,26) = 4.7, *p* = 0.013, ηp^2^ = 0.15]. The interaction analyses showed a main effect of Sex in P region [*F*(1.27) = 6.7, *p* = 0.010, ηp^2^ = 0.20; males (*X* = 1.2 mV, SD = ±0.70), females (*X* = 3.6 mV, SD = ±0.63)] and in O region [*F*(1,27) = 10.5, *p* = 0.003, ηp^2^ = 0.28; males (*X* = 1.8 mV, SD = ±0.87), females (*X* = 4.5 mV, SD = ±0.76)], but not in C region (*p* > 0.05). No other main effect nor interactions were found.

##### P300: time window from 350 to 550 ms after the response image onset

Again, a significant main effect of Sex [*F*(1,27) = 5, *p* = 0.034, ηp^2^ = 0.16] was found, with greater amplitude in female participant (*X* = 3.4 mV, SD = ±0.64) compared to male participants (*X* = 1.3 mV, SD = ±0.71); a main effect of Caudality [*F*(2,26) = 23.9, *p* < 0.001, ηp^2^ = 0.65], greater activity in P region (*X* = 3.1 mV, SD = ±0.52) and in O region (*X* = 3.2 mV, SD = ±0.66) than in C region (*X* = 0.6 mV, SD = ±0.53) was registered. Also, a main effect of Criteria [*F*(1,27) = 9.9, *p* = 0.004, ηp^2^ = 0.27] was found, with a lower amplitude in Position trials (*X* = 1.8 mV, SD = ±0.49) than in Color trials (*X* = 2.8 mV, SD = ±0.52).

**FIGURE 9:**
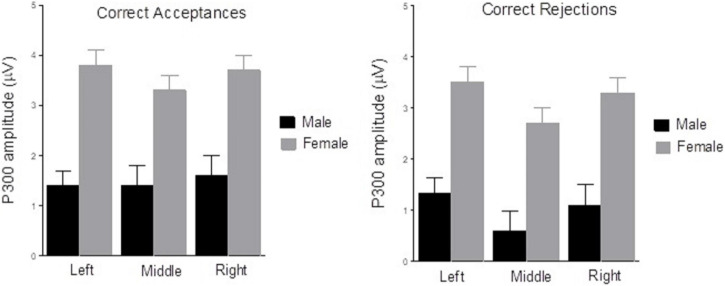
Mean of P300 amplitude (in mV) of Hits **(left graph)** and Correct Rejections **(right graph)** as a function of Laterality, Left (C3, P3, O1), Middle (Cz, Pz, Oz), and Right (C4, P4, O2), and Sex (male, female), in the Retrieval phase. There was a main effect of Sex (*p* < 0.050). Laterality main effect was found only in Correct Rejections, *p* = 0.002. There were significant differences between Left and Middle laterality, *p* = 0.001; and Middle and Right laterality, *p* = 0.004.

Sex *X* Caudality interaction effect reached also significance [*F*(2,26) = 4.5, *p* = 0.02, ηp^2^ = 0.26]. As in the previous phases (encoding and maintenance), the interaction analysis showed a main effect of Sex in P region [*F*(1.27) = 6.2, *p* = 0.02, ηp^2^ = 0.19; males (*X* = 1.8 mV, SD = ±0.77), females (*X* = 4.4 mV, SD = ±0.69)] and in O region [*F*(1,27) = 7.6, *p* = 0.01, ηp^2^ = 0.22; males (*X* = 1.4 mV, SD = ±0.98), females (*X* = 5.1 mV, SD = ±0.88)], but not in C region (*p* > 0.05). Finally, Decision Type *X* Laterality interaction effect was significant [*F*(2,26) = 3.6, *p* = 0.035, ηp^2^ = 0.12]. Further analyses of the interaction showed that the Laterality main effect was found only in Correct Rejections trials [*F*(2,26) = 8.3, *p* = 0.002, ηp^2^ = 0.39]: as can be seen in Figure 9, there were significant differences between Left and Middle laterality [*F*(1,27) = 13.5, *p* = 0.001, ηp^2^ = 0.33]; and Middle and Right laterality [*F*(1,27) = 9.7, *p* = 0.004, ηp^2^ = 0.26], with lower activation of central sites in all cases. Although Decision Type × Laterality × Sex interaction was not found (*p* > 0.05), complementary *T* test analysis revealed a main effect of Decision Type only in middle axis (Cz, Pz, and Oz) for the male group [*t*(12) = 2.1, *p* = 0.041], with decreased amplitude for correct rejections compared to hits. No other main effect nor interactions were found (*p* > 0.05).

## Discussion

The main goal of our study was to identify the underlying electrophysiological correlates of performance in an allocentric spatial recognition, exploring any sex-related pattern. In order to do so, a computerized spatial recognition memory task with viewpoint manipulation in a complex environment was used. It was based on the Almeria Spatial Memory Recognition Test (ASMRT, Tascón et al., 2017) alongside the ERP technique.

### Behavioral Findings

Our results showed that performance was similar in both sexes. Otherwise, reaction times suggest that correct rejections implied greater cognitive demand, as suggested before (Coluccia and Louse, 2004), because they took significantly longer time than correct acceptances (successful responses to correct items). This could be related to visuo-spatial working memory load, according to Bosco et al. (2004). Previous studies with ASMRT showed that spatial differences between male and female participants arose only in certain difficulty levels, which generally demanded the encoding of three colored boxes (Tascón et al., 2017).

After the memorization phase, a second picture of the room was presented, but this time with a single-colored box and a perspective rotated from the first picture. Hence, participants were not only demanded to form a mental map of the room and memorize the location of all three colored boxes in relation to the environment, but also consider unannounced changes in perspective in order to match the new viewpoint. This shift demanded participants to imagine the room from novel viewpoints, thus requiring knowledge of the relationships between stimuli for an optimal orientation. This allocentric processing is medial temporal lobe dependent (Iaria et al., 2009) and, particularly relies on the hippocampus, a structure well-known for its involvement in spatial performance. Hence, damage to this structure is related to impaired spatial orientation performance (Astur et al., 2002; Maguire et al., 2001) and there are pronounced hippocampal activations in viewpoint rotation tasks (Lambrey et al., 2011). In addition to this, the hippocampus is known to have functional differences between males and females (Koss and Frick, 2016), such as a more right-lateralized activation in men (Persson et al., 2013). This sexual dimorphism trend extended to other structures related to spatial processing (Kaiser et al., 2008; Semrud-Clikeman et al., 2012).

As a consequence, the way males and females engage in strategic processing of spatial information can differ. Certain studies identified that sex could modulate the understanding of maps, and that, specifically, males tended to rely more on Euclidean or abstract references, while females were more concrete and landmark oriented (Dabbs et al., 1998; Sandstrom et al., 1998; Nazareth et al., 2018).

Our behavioral results did not find reliable sex differences in contrast to the general trend of male advantage in visuospatial demands (Voyer et al., 2017). Males and females performed similarly in parameters like correct responses or latencies, differing from previous studies using the ASMRT (Tascón et al., 2017). Nevertheless, there are important differences between the original ASMRT and the version adapted in this study that might explain these findings. The increase in the number of trials compared to the original task could produce a training effect. Note that enhanced number of trials (128 presentations of the room from multiple perspectives) increases familiarity with the environment. Participants have more opportunities to learn the spatial layout. A previous study by Livingstone-Lee et al. (2014) showed that spatial training in both allocentric and egocentric strategies can eliminate sex differences, and our participants were continuously exposed to allocentric demands in the same environment. Regarding this, increased familiarity with the environment can also reduce this effect, as explained by Nori et al. (2018). In addition, a memory image was followed by ten recognition images (trials) in the original task, whereas EEG adaptation demanded a correspondence of 1:1 between memory and recognition trials, thus, allowing for more encoding opportunities.

Furthermore, participants performed better in color trials versus position trials. Using the same images and procedure, color trials demanded to remember the color of the boxes and no positions. The “color” condition was a control of the “position” condition: same images as in “position trials” but demanding more basic processes (color recognition). This implies lower cognitive demands and potentially different underlying mechanisms, confirmed in the ERP findings.

The WMC was also assessed using the Change Localization Task, which proved to be a useful tool to disclose WMC cognitive differences in multiple previous studies (Johnson et al., 2013; Noguera et al., 2019; Castillo et al., 2020; Fernández et al., 2021). In our study, higher WMC was related to better rejection rates in position trials regardless of the sex factor. Visuospatial working memory and spatial orientations were proven to be related (Bergmann et al., 2016), and higher working memory capabilities were associated to a superior performance in a virtual spatial orientation task (Castillo et al., 2020).

### Electrophysiological Findings

Despite the fact that there was no difference on general performance between males and females, the ERPs showed a reliable sex difference across all three phases of memory process—encoding, maintenance and retrieval—regardless of decision type or criteria. Could these sex electrophysiological differences be due to different ways of processing spatial information, or due to different ways of looking at it? In this regard, although sex differences in the visual system, including visual perception, have been widely described (see Vanston and Strother, 2017, for a review), our results did not show reliable differences between males and females, due to criteria, in early ERP components (neither P100, N100 nor P200). Nonetheless, higher amplitudes in the female group were observed in positive early waves compared to the male group, in all phases of the memory process. In contrast, the male group showed higher amplitudes in negative early components, thought to be related to attentional control mechanisms (Vanston and Strother, 2017).

#### Encoding

The N200 component arose 250 ms after the memory image onset. This negative ERP component has been commonly related to encoding of visuospatial information and selective attention in posterior sites (Corbetta and Shulman, 2002; Silver et al., 2005), reflecting top-down modulations related to higher cognitive functions (Lithfous et al., 2014). The higher amplitude in this time window, is usually associated with an increase in cognitive control (Folstein and Van Petten, 2007; Potts, 2011). Our results revealed a reliable higher voltage drop in the male group than in the female group, in the three phases of memory process, which suggests that males recruited more cognitive control mechanisms compared to females, at early steps of processing.

The N200 neural generators has been commonly located in the dorsal anterior cingulate cortex (ACC) and in the midcingulate cortex (MCC) (Huster et al., 2010), both regions have relevant neural connections with cortical parietal areas. These regions are involved in attentional control mechanisms (Huster et al., 2010, 2014). In addition, recent research suggests that the medial temporal lobe (MTL) could be a key source of this early component, involving hippocampal formation (Raynal et al., 2020).

Immediately later, the P300 component appeared. This ERP has been shown reflecting selective attention, stimulus evaluation and categorization (Lithfous et al., 2014; Carmona et al., 2020b). Its amplitude can be modulated by the allocation of attentional resources (Kok, 2001). The amplitude of P300 was significantly higher in females than in males, supporting the differences showed in several research (Roalf et al., 2006; Steffensen et al., 2008). Roalf et al. (2006) suggested that differences in hemispheric asymmetry could provoke the greater P300 amplitudes exhibited by females compared to males. Female brains are commonly less lateralized compared to those of males (Kolb and Whishaw, 1996). However, the way in which the hemispheric asymmetry can affect the amplitude of this component is not yet established.

The temporoparietal junctions have been shown as one of the different generating sources of the P300, along with regions such as the insular cortex, the inferofrontal cortex and the MCC (Huster et al., 2010, 2014). Also, the MTL has been identified as neural generators of this component, specifically, the hippocampal regions, suggesting the recruitment of the subiculum and the hippocampal areas (Ludwig et al., 2009). Otherwise, Kosmidou et al. (2015) suggest that the differences in attentional control mechanisms between males and females could be the consequence of the different brain lateralization exhibited by each sex that could imply the differential contributions of generating sources. In this regard, the implication of the hippocampal formation in the generation of the N200-P300 tandem is relevant in our task since electrophysiological sex differences are found in both components. The hippocampal functional differences between males and females and the greater lateralization exhibited by men (Persson et al., 2013; Koss and Frick, 2016) could help to explain our results. In addition, sexual dimorphism has been found in hippocampal subfields, specifically in the subiculum (Van Eijk et al., 2020), also described as a P300 generator (Ludowig et al., 2010). Moreover, neuroanatomical sex differences have been found in the MCC and they have been related to differences in attentional control (Huster et al., 2010, 2014).

After a second, the NSW begins. This sustained wave for several seconds, is usually related to encoding and maintenance of visuospatial information (Ruchkin et al., 1992; Woodman and Vogel, 2008; Morgan et al., 2010). Research suggests that the amplitude of this negative wave is sensitive to working memory load; therefore, when memory load increases, wave amplitude increases (Morgan et al., 2010). The increase in amplitude of the NSW can also be explained in terms of sustained cognitive demand over time (Borragán et al., 2017) or greater task difficulty (Rösler et al., 1997). The NSW has also been related to the information path of what and where, with lower voltage for visual than for spatial or complex stimuli (Woodman and Vogel, 2008; Luria et al., 2010). According to these accounts, our results showed that Position trials elicited NSW with greater amplitude compared with color trials. It should also be noted that males and females showed different NSW pattern in position trials: this wave emerged in parietal and occipital regions in both groups, and in central sites only in the female group. While in color trials, the NSW was similar in both groups, active in occipital region, and null in central and parietal regions. In addition, the NSW in the female group showed a significantly higher amplitude in position trials than in color trials. Based on the aforementioned research, these results would indicate that the position trials required more cognitive demand, or implied greater working memory load, than those of color for females. By contrast, the NSW amplitude in the male group was similar in both criteria and lower than in the female group. Also, in parietal region reliable sex-related differences in NSW amplitude were found. Specifically, it could be argued that the emergence of NSW in parietal regions, and in central regions in some cases, was an index of task difficulty or higher cognitive demands (related to position trials), as occipital regions was active regardless of criteria.

The NSW neural generators in the memory task remain unclear. Recent research suggests that this sustained activity could be due to the synchronization between the MMC and the bilateral parietal regions, it explained as the MCC deactivation and the bilateral parietal activation (Liu et al., 2018). Again, the MCC appears as key region to promote cortical activity, in this case by deactivation that facilitate the parietal activation. The different morphology exhibited by males and females in the MCC could contribute to the electrophysiological differences. Additional research has found evidence of the MTL involvement in this negative sustained activity pattern, although without describing the involvement of specific areas (Axmacher et al., 2007).

#### Maintenance

Over a short delay between the memory image and the retrieval, two ERP components were elicited, N200 and P300, as reflect of maintenance in working memory (Polich, 2007). Again, as in the encoding phase, higher amplitude in the N200 and lower amplitude in the P300 were exhibited by the male group compared to the female group. The greater attentional control resources triggered, reflexed by the higher N200 amplitude by men, could be interacting with the P300. The decreased in P300 amplitude is commonly related with greater cognitive demands or greater amount of attention resources triggered (Polich, 2007).

#### Retrieval

Several researchers have argued that working memory load and task difficulty also modulate the amplitude of the N200 component, in a way that higher working memory loads or difficulty elicit larger N200 amplitude (Missonnier et al., 2003). According to this account, in the retrieval phase, significantly higher amplitudes were registered in position trials compared to color trials, reflecting at electrophysiological level the results at behavioral level.

Regarding to the P300, memory updating processes has been related to this component, referred to the updating of a context-environment model previously registered in memory, through an attention-driven process of comparison between the information kept in working memory (mental representation) and the information that has just been displayed (Donchin and Coles, 1988; Polich, 2007). This attention-driven process is influenced by working memory load and task difficulty (Scharinger et al., 2017). Lower amplitudes in this component have been associated with higher memory loads (Kok, 2001; Scharinger et al., 2015, 2017). In addition, when the manipulation of the information in working memory is required, the decreased in P300 amplitude has been related to the internal allocation of controlled attention (Watter et al., 2001; Scharinger et al., 2017). Our results showed, again, a lower amplitude of P300 in the male group compared to that of females. In this phase of memory processing, the lower amplitude exhibited by males suggests that cognitive demands in this attention-driven comparison process were greater compared to females. The same wave pattern was observed when comparing position and color trials, reflecting the greater difficulty to respond to position trials.

With regard to the Decision type, lower P300 amplitudes were found in Correct rejections trials compared to Hits trials, although differences were significant only in the men group, in the middle axis. This result could explain the better performance of males in correct rejections compared to that of females. However, more research is needed to further explore this account.

### Conclusion

In summary, the time course of the differences due to sex found in the electrophysiological events analysed, revealed the differential use of cognitive resources at different time intervals, with similar general performance in the task. It could correspond to electrophysiological correlates of different strategic processes triggered by males and females. Males activated more attentional control mechanisms at early stages of processing (N200), followed by greater internal allocation of attentional resources and memory updating processes (P300) than females, in the three phases of memory process. While females exhibited longer cognitive effort, sustained for several seconds, in later steps of encoding process (NSW).

Oscillatory analysis could help to clarify the underlying process to sex differences revealed in our study. An analysis of ERP in the time-frequency domain would be a complementary source of results to time domain that would offer useful and valuable information.

## Data Availability Statement

The datasets presented in this study can be found in online repositories. The names of the repository/repositories and accession number(s) can be requested to the corresponding author.

## Ethics Statement

The studies involving human participants were reviewed and approved by the University of Almería Ethical Committee and fulfills the requirements of the European Communities Council Directive 2001/20/EC. The patients/participants provided their written informed consent to participate in this study.

## Author Contributions

JC: investigation, writing – original draft, writing – review and editing, visualization, methodology, and conceptualization. IC: investigation, writing – original draft, writing – review and editing, formal analysis, and data curation. SC: writing original – draft, writing – review and editing, and validation. SF: writing original – draft, validation, methodology, and resources. JJO: writing original – draft, validation, conceptualization, methodology, and resources. JMC: project administration, funding acquisition, conceptualization, methodology, writing original – draft, writing – review and editing, supervision, and validation. All authors contributed to the article and approved the submitted version.

## Conflict of Interest

The authors declare that the research was conducted in the absence of any commercial or financial relationships that could be construed as a potential conflict of interest.

## Publisher’s Note

All claims expressed in this article are solely those of the authors and do not necessarily represent those of their affiliated organizations, or those of the publisher, the editors and the reviewers. Any product that may be evaluated in this article, or claim that may be made by its manufacturer, is not guaranteed or endorsed by the publisher.
